# Revisiting gendered parenting of adolescents: understanding its effects on psychosocial development

**DOI:** 10.1007/s12144-022-03536-7

**Published:** 2022-08-10

**Authors:** Cassandra K. Dittman, Madeline Sprajcer, Emma L. Turley

**Affiliations:** 1 Central Queensland University, Locked Bag 3333, 4670 Bundaberg, DC, QLD Australia; 2 Parenting and Family Support Centre, School of Psychology, The University of Queensland, St Lucia, QLD Australia; 3 Appleton Institute, Central Queensland University, Norman Gardens, QLD Australia

**Keywords:** Adolescents, Gender development, Gender roles, Parenting, Gendered parenting, Review

## Abstract

**Introduction**: Today’s adolescents are growing up in a unique sociocultural climate in which gender issues are highly prominent. Alongside new ways of understanding gender identity, there are persistent gender disparities in social, health and mental health outcomes despite increasingly egalitarian views and a significant public focus on sexual assault and gender-based violence. Given gender-differentiated outcomes emerge during adolescence, it is critical to revisit factors influencing adolescent gender development. It has been argued that gendered parenting, reflected in differences in parenting attitudes and behaviors directed towards boys and girls, influences gender development. While numerous studies have examined gendered parenting with children, there has been no previous synthesis of gendered parenting of adolescents. **Method**: The current narrative review presents an overview of research into gendered parenting of adolescents, including parental modelling, gendered environments, and specific parenting practices, and draws together the available research on how it impacts adolescents. Gendered parenting is also examined in the context of LGBTQI + and gender non-conforming adolescents. **Results**: There is limited research investigating the presence of gendered parenting of adolescents, and even less assessing its impact on adolescent psychosocial outcomes. The available literature suggests that there may be effects of gendered parenting on adolescents, particularly on their gender role attitudes and gender-typed behaviors. **Conclusions**: Future work is needed to better understand how gendered parenting of adolescents manifests in the family home. In addition, research is needed to examine the longitudinal impact of gendered parenting, particularly within non-traditional families, and across a range of sociocultural contexts.

Gender socialisation begins at birth, and gender identity and expression in adolescence is shaped by and extends upon childhood characteristics and experiences (Galambos et al., 2009). Yet, the dynamic nature of adolescence means that there is still considerable advancement during this period in how young people perceive, understand, and express their own and others’ gender, and in their values and attitudes about gender generally (Kagesten et al., 2016). Further, gender is likely to affect how adolescents manage the hallmark transitions of this developmental period including the biological changes brought about by puberty, changes in peer and intimate relationships, increasing autonomy from parents and family, and planning their future academic and employment pathways (Blum et al., 2017; Perry & Pauletti, 2011). While external influences, like peers, teachers, and the media, become increasingly important in an adolescent’s life, many studies point to the central and continuing role of parents in adolescent development (Ali et al., 2015; Galambos et al., 2003). Yet, the role of parents in the gender development of adolescents specifically has been relatively unexplored (Galambos et al., 2009), particularly in comparison to theory and research highlighting parental influence on children’s gender development (Endendijk et al., 2016; Lytton & Romney, 1991; McHale et al., 2003; Spears-Brown & Tam, 2019).

Gendered parenting is the term that has been used to describe the explicit and implicit messages that children receive from parents regarding how boys and girls should and should not behave (Mesman & Groeneveld, 2018). Gendered parenting is argued to be reflected in the specific parenting practices, rather than broad parenting styles, that directly or indirectly communicate parental expectations about child behavior based on their biological sex and therefore contributes to a child’s gender development (Mesman & Groeneveld, 2018). While numerous studies and several reviews have examined gendered parenting with children (e.g., Endendijk et al., 2018; Morawska, 2020), there has been far less attention directed at the issue of gendered parenting of adolescents.

Thus, this article is a narrative review that aims to provide a synthesis of research examining gendered parenting in adolescence. The paper has three broad objectives. Firstly, the paper aims to provide a rationale for revisiting gendered parenting of adolescents by providing a discussion of the contemporary sociocultural context within which current adolescents are undergoing gender development. Secondly, the paper will provide a review of what is known about gendered parenting and its effects on adolescent psychosocial development. This will include the effects of gendered parenting related specifically to gender development (e.g., gender-typed behavior, gender roles, gender attitudes), but also to psychosocial outcomes more broadly (i.e., behavioral, emotional, and social functioning). Research related specifically to gender non-conforming and LGBTQI + adolescents will also be briefly reviewed here. To help contextualise this discussion of gendered parenting and its effects on adolescents, a brief overview of gendered parenting of children will be provided first. Thirdly, the paper aims to identify future directions for research into gendered parenting of adolescents.

## The sociocultural impetus for research into parenting and gender development in adolescents

The 1970s saw a major transformation in our conceptualisation of sex and gender, with the advancement of the notion that social factors play a larger role in gender identity and gender roles than biology (Zosuls et al., 2011). This led to a focus in the literature across the 70 and 80 s on parents’ differential socialisation of girls and boys, and resulting gender role attitudes (Zosuls et al., 2011). While research into parental influences has continued to be a feature of gender development research since then (Endendijk et al., 2018), there are several sociocultural forces that mean that it is timely to revisit and reinvigorate research into parenting and gender development in adolescents.

Indices of gender equality (e.g., female workforce participation, female educational attainment, representation of women in politics and leadership) have significantly improved in the past five decades (Boehnke, 2011; Thijs et al., 2017), and evidence suggests that gender beliefs have become increasingly egalitarian (Scarborough et al., 2019; Thijs et al., 2019). Yet, gender inequality is still a significant feature of our society, particularly in low- and middle-income countries (LMIC). Sociological theory and research emphasise that intergenerational transmission of traditional gender-role attitudes contribute to sustained gender inequalities (Heise et al., 2019), underscoring the need to investigate parents’ role in gender development. Further, adolescence is a time when gender-differentiated patterns in social, health and mental health outcomes start to emerge, particularly in LMIC (LMIC; Kagesten et al., 2016; Patton et al., 2009). Adolescent girls are more likely than their male counterparts to report poorer self-rated health (Torsheim et al., 2006) and suicidal ideation and anxiety (Biswas et al., 2020), experience forced sexual initiation (Moore et al., 2007), and are at risk of maternal mortality (Patton et al., 2009). Over their life, they are more likely than their male counterparts to experience intimate partner physical and/or sexual abuse (Australian Bureau of Statistics, 2016; United Nations, 2020), live in extreme poverty (Manandhar et al., 2018), develop a disability (*World Report on Disability*, 2011), and experience depression and anxiety (Riecher-Rössler, 2017). Adolescent males have higher mortality rates, reflecting more deaths from traffic accidents and interpersonal violence (Patton et al., 2009), and are at greater risk of alcohol and drug use (Mokdad et al., 2016) and suicide (Hee Ahn et al., 2012).

While global disparities in outcomes between male and female youth are clearly concerning, public attention has recently focused on issues of gender-based violence and sexual assault and discrimination (Oldfield & McDonald, 2021). There are examples of this across the world, including in Australia where a cluster of recent high profile sexual assault cases in Federal Government, and several suburban intimate partner homicides, have attracted outrage and extensive media coverage. The worldwide #MeToo social justice movement, in particular, has brought these issues to the forefront of public attention (Caputi et al., 2019; Lazard, 2020). Further, the unprecedented social impact of the COVID-19 pandemic has included a trend toward a return to traditional gender roles, with women being more likely to have work disrupted by childcare, to lose their jobs and/or to work in essential jobs where they are exposed to infection (Carli, 2020).

As well as this unique sociocultural backdrop, adolescent gender development is occurring in the context of new ways of thinking about gender. Today’s adolescents are among the first to be exposed to a shift from conceptualising gender identity as dichotomous categories of male and female (i.e., a gender binary) to the notion of multiple gender identities (Cheung et al., 2020; Monro, 2019). While prevalence rates are difficult to determine because of evolving terminology, research indicates a growth in the proportion of individuals who self-identity as transgender or non-binary, with a higher prevalence of transgender or non-binary identities among older adolescents and young adults (Nolan et al., 2019). Thus, issues of gender identity are highly visible to today’s adolescents who have a better awareness of gender identities such as genderfluid, genderqueer, transgender, and non-binary (Bragg et al., 2018). For instance, a U.K. qualitative study exploring 12 to 14-year-old students’ views on gender diversity and equality across five schools revealed that adolescents have a wide vocabulary for discussing gender identity and expression, with 23 different terms for gender identity used by study participants (Bragg et al., 2018). Further, data from a large, nationally-representative 2018 U.S. survey indicated that 35% of U.S. adolescents aged 13 to 17 years reported that they personally knew someone who prefers to be referred to using gender-neutral pronouns, compared to 25% of adults aged 22 to 37 years and 16% of adults aged 38 to 53 years (Pew Research Center, 2019).

Thus, today’s adolescents are growing up in a world in which gender issues are highly salient and gender is being conceptualised in a way that it never has before. This provides a unique and new sociocultural context for investigating gender development in adolescence. Importantly, given the central role of parents in gender development specifically and adolescent development and psychosocial wellbeing generally, research is needed to examine how gendered parenting plays out within the current sociocultural context, how it influences adolescent gender-typed behavior and attitudes, and how this affects psychosocial outcomes for adolescents.

## Gendered parenting during childhood and its effects on children

Gender development has been considered from evolutionary (Trivers, 1972), cognitive-developmental (Kohlberg, 1993), and information processing (Martin & Halverson, 1981) theoretical perspectives. However, social learning models (Bussey & Bandura, 1999; Mischel, 1996) provide the most direct account of the role of parents and families in children’s acquisition of gender roles (see Endendijk et al., 2018 for an integrative review of family processes in gender development). Social learning accounts of gender development view gendered parenting as an implicit rather than an overt parenting practice, with parents transmitting messages to children about gender-appropriate behavior directly and indirectly (Mesman & Groeneveld, 2018). Specifically, social learning approaches argue that parents contribute to child gender development through modelling and imitation of gender roles, and via direct and vicarious reinforcement and punishment of gender-typed behavior. According to Endendijk et al. (2018), parents reinforce gender-typed behaviour in a number of ways, including through the creation of a gendered environment, parent-child communication, and differential parenting practices and behaviors (Endendijk et al., 2018).

When it comes to parental modelling influences, parental gender roles in the home transmit direct messages to children about appropriate roles for men and women and create opportunities for the transmission of gender role attitudes (Sutfin et al., 2008). In homes where mothers take a more traditional feminine role by completing more household tasks and undertaking more childcare duties than fathers, girls are more likely to demonstrate stereotyped gender role attitudes (Halpern & Perry-Jenkins, 2016). In contrast, when families exhibit equal division of labour in the home, children can think more flexibly about gender roles (Fulcher et al., 2008) and when fathers perform more childcare duties, their children demonstrate fewer gender stereotypes (Turner & Gervais, 1995). Thus, one of the major outcomes of parental modelling of gender roles are children’s own attitudes toward gender, particularly since parents who subscribe to stereotyped ideas about gender transmit these attitudes to their children (Epstein & Ward, 2011; Fulcher et al., 2010).

In addition to parental modelling of gendered behavior and attitudes, a gendered home environment can be created by parents in terms of the toys, games, books, media, and other commercial products that they choose for their children (MacPhee & Pendergast, 2019; Leaper & Farkas, 2015; Axinn et al., 2011). Even despite the advent of more egalitarian attitudes in Western culture, children’s bedroom décor, toys, and other contents still tend to be based on traditional gender norms (MacPhee & Pendergast, 2019; Rheingold & Cook, 1975), with heterosexual parents more likely to provide different toy choices for boys and girls in terms of type, style, and colour (Boe & Woods, 2018), and cross-gender toys viewed as less desirable by parents than same-gender or gender-neutral toys (Kollmeyer et al., 2018). Toy availability has been found to influence young children’s toy preferences (Eisenberg et al., 1985), with Boe and Woods (2018) reporting that infants display gender-typical toy preference at 12.5 months old when exposed to gender-typical toys in the home. In terms of the effects on children’s psychosocial development, availability of gender-typical toys and activities are likely to influence children’s play (Morawska, 2020). Correlational studies have demonstrated that boys engage in more exploratory play with objects such as blocks, and girls show a preference for symbolic play, for instance playing with dolls (Suizzo & Bornstein, 2006; Fagot, 1974). Though more research is needed, given the importance of play in children’s social and cognitive development, play that is based around narrowly defined gender expectations may reinforce traditional gender norms and attitudes (Friedman et al., 2007) and limit exposure to, and positive experiences with, gender atypical activities (Reilly & Neumann, 2013).

Differences in parents’ language interactions with boys versus girls may also influence psychosocial outcomes for children (Morawska, 2020). In a longitudinal study with young children by Adams et al. (1995), mothers used more emotional talk with their daughters but were less likely to attribute positive emotions to girls than to boys, and consequently girls were able to draw on a wider variety of emotional words from their vocabulary than boys. This was supported by a recent systematic review that found that gendered emotional socialisation of young children was associated with differences in child outcomes in displays of emotion and children’s use of emotion language and words (Morawska, 2020). Differences in content and topics of parent-child conversations have been found to produce different outcomes for boys and girls. Pruden and Levine’s (2017) longitudinal study reported that parents used more spatial words when they were communicating with boys and as a result, boys produced more spatial language than girls in their own communication. Similarly, Flannagan and Ward’s (1996) study reported that mother-son conversations focused on learning-related topics while mother-daughter conversations were more likely to focus on socialising and discussions about other individuals, which was then reflected in the children’s own talk. Parents send implicit messages to children by focusing on specific gender-dependant content in conversations, which may have implications in terms of children’s understanding about the importance of these types of activities for them (Morawska, 2020).

Parental attitudes about gender may influence the parenting practices in terms used with boys versus girls, particularly in terms of levels of discipline and control. Research has found that mothers responded less negatively to boys’ disruptive behavior than girls’ but are less encouraging of their son’s prosocial behaviors (Mesman & Groeneveld, 2018; Martin & Ross, 2005). Endendijk et al.’s (2018) longitudinal study found that fathers with stereotyped gender attitudes used more physical control with their sons than their daughters, which in turn was associated with greater aggressive behavior among these boys. This suggests that greater use of physical control for male children models the use of aggressive behavior and encourages the development of this behavior in boys (Morawska, 2020). Thus, gendered parenting is present in childhood and may result in differing outcomes for children depending on their gender.

## Gendered parenting during adolescence and its effects on adolescents

Major reviews and meta-analyses into the influence of parents on gender development in childhood tend to make broad assumptions about the generalisability of their findings to the adolescent period (Endendijk et al., 2016; Lytton & Romney, 1991; McHale et al., 2003; Spears-Brown & Tam, 2019), and research specifically examining gendered parenting during adolescence is limited (Galambos et al., 2009). In this section, the social learning account of gender development and the conceptualisation of gendered parenting of children proposed by Endendijk et al. (2018) will be used to guide the review in terms of what is known about gendered parenting of adolescents and its effects on gender development specifically, and psychosocial outcomes more broadly.

### Parental modelling

The minimal research with adolescents suggests that parents continue to serve as important models of gender attitudes and roles throughout the adolescent period (Galambos et al., 2009). Research indicates that time with parents is gendered, in that mothers are more likely to spend time with their adolescent daughters, and fathers with their adolescent sons (Tucker et al., 2003). This exposure to male versus female role models in the home appears to influence preferred interests and activities. Longitudinal work from early to late adolescence indicated that adolescent time spent with mothers was linked with engagement in stereotypically feminine interests and activities (e.g., dance, music, handicrafts, reading, walking), and vice versa for fathers and masculine interests and activities (e.g., sports, watching television, maths, science; McHale et al., 2009).

An important mechanism by which gendered parenting is likely to affect outcomes for adolescents is through the intergenerational transmission of gender role attitudes. In a cross-cultural systematic review, Kagesten et al. (2016) found that parental attitudes were reliably associated with the gender role attitudes of young adolescents, particularly among girls (see also Tenenbaum & Leaper, 2002). This finding is supported by longitudinal Australian research drawing on nationally representative data from 1,806 14 to 15-year-old adolescents and their parents (Perales et al., 2021) found that both mothers and fathers who espoused egalitarian gender role attitudes were more likely to have adolescents who supported egalitarian attitudes (and vice versa for traditional attitudes). Interestingly, where parents held opposing gender role beliefs, the influence of the parent with the traditional attitudes was overshadowed by the egalitarian attitudes of the other parent. Longitudinal research from the U.S. indicated that for both boys and girls having a male sibling in combination with parents who endorsed traditional attitudes pushed adolescents to hold more traditional attitudes by late adolescence (Crouter et al., 2007).

Overall, research is needed to investigate the effects of intergenerational transmission of gender attitudes on adolescent development, including effects on career choice, future family roles, and any gender disparities in physical and mental health. Further, little is known as to *how* these gender attitudes are conveyed to adolescents. Within societies that hold more egalitarian values, parents are unlikely to report that they hold gender-stereotypical beliefs or that they communicate these to their children (Mesman & Groeneveld, 2018). One strategy for eliciting implicit parental gender stereotypes may be to observe naturalistic parent-adolescent interactions in response to stimuli (e.g., music videos, TV shows) containing stereotypical and counter-stereotypical characters and events, mirroring research with younger children that has revealed gendered patterns in parental discussions during storybook reading (Mesman & Groeneveld, 2018).

### Gendered environment

Gendered environments created by parents during childhood are likely to continue into adolescence, with ongoing reinforcement of gender roles through exposure to media, social experiences, the selection of gifts and clothes, and allocation of chores and family privileges (Galambos et al., 2009), although this may depend on constellation of siblings within the family (e.g., same-sex or opposite-sex siblings; Tucker et al., 2003) and cultural factors (McHale et al., 2005). Parents also continue in their role as gender gatekeepers, encouraging their adolescents toward involvement in particular interests and activities and providing resources and opportunities consistent with parental gender role beliefs, either traditional or egalitarian (McHale et al., 2009).

According to the work of Eccles and her colleagues, the beliefs that parents hold for their children influence parent-child interactions, particularly the extent and provision of resources and opportunities, which in turn affects a child’s motivation and achievement (Eccles & Harold, 1991; Wigfield & Eccles, 2000). Thus, the extent to which the home environment is gendered may have longer-lasting effects on adolescents, shaping their self-concept and personal evaluations of skills in a particular domain. For example, research has found that adolescent boys’ self-concept is greater for sports, physical appearance and sometimes mathematics, while girls evaluate their self-concept in relation to strengths in verbal/reading ability and social relationships (Jacobs et al., 2002; Klomsten et al., 2004; Watt, 2004). There is little data on the long-term implications of parent-influenced gendered interests, activities, and self-concept among adolescents. One longitudinal study with young adolescents found that girls’ time with mothers is associated with better language arts/English grades two years later, and girls’ time spent with fathers’ positively predicted mathematics grades (McHale et al., 2004). Research is needed to investigate whether parent-influenced gendered activities and interests influence opportunities and decisions currently, such as subject choice and achievement at secondary school, and in the future, such as engagement in further education, occupational pathways and family formation and roles (Galambos et al., 2009).

### Parenting Practices

Like the evidence base with younger children, research with parents and adolescents suggests that effective discipline and limit-setting, and parental support and warmth continue to be important in the healthy development of adolescents (Galambos et al., 2003; Pinquart & Gerke, 2019). Research examining gender differences in the parenting of adolescents has focused on child gender as a moderator of the association between parenting and adolescent outcomes; that is, whether the relationship between parenting and adolescent outcomes differ depending on child sex (Pinquart, 2017a). For instance, an early study by Inoff-Germain et al. (1988), which compared families of 30 adolescent boys and families of 30 adolescent girls, found differential associations between parental behavior and adolescent behavior based on gender, with maternal and paternal control associated with aggression among boys, but not girls. More recently, cross-national and meta-analytic research has indicated that child gender does not moderate the relationship between parenting and adolescent externalising problems (N = 1,435 studies; Pinquart 2017a) and delinquency (N = 161 studies; Hoeve et al., 2009), school conduct and psychological adjustment (N = 9 studies across 12 countries; Ali et al., 2015), academic achievement (N = 308 studies; Pinquart, 2016) or prosocial behaviour (N = 9 countries; Putnick et al., 2018), with some evidence that there is a stronger association between parental warmth and internalising symptoms among girls compared to boys (Pinquart, 2017b).

While there is an abundant evidence base for the effects of child gender on the impact of parenting on adolescents, this research does not examine whether parents differentially apply certain parenting practices to their female adolescent children in comparison to their male adolescent children. Indeed, very little research has investigated differential application of parenting practices to adolescent boys versus girls. Only two out of 126 studies in the meta-analysis on differences in parental discipline and control of boys and girls by Endendijk et al. (2016) involved children aged over 12 years. One of these studies found no association between adolescent gender and observer ratings of maternal and paternal use of high levels of monitoring and control (Gunnoe et al., 1999), while the other found that mothers of boys were more likely to use manipulative and controlling behavior in interactions than mothers of girls (Inoff-Germain et al., 1988). Thus, there has been very few attempts to document differential parental treatment of adolescent girls and boys, making it difficult to know the extent to which this occurs and whether it has any impact on outcomes for adolescents.

For adolescents, an important parenting construct to consider is parental monitoring and supervision (Kerr & Stattin, 2000), given the association between these parenting practices and outcomes for adolescents, including adolescent conduct problems (Racz & McMahon, 2011; Walters, 2019) and depression (Yap et al., 2014). Several studies have found that girls are more monitored than boys, parents have greater knowledge of girls’ whereabouts and activities than boys, and girls spontaneously disclose more to parents than boys (Racz & McMahon, 2011). However, one study involving both between- and within-family comparisons suggest that this may change with birth-order, with second-born sons being more highly monitored and having lower decision-making input than first-born daughters (Bumpus et al., 2001). Differential parental monitoring may also be dependent upon parental gender-role attitudes, with one study finding that mothers’ traditional gender attitudes affected monitoring and autonomy-granting of daughters more so than sons (Bumpus et al., 2001).

Overall, the available research indicates that gendered parental monitoring may not play a role in gender differences in involvement in antisocial behavior (e.g., Storvoll & Wichstrøm 2003) and depression (Riecher-Rössler, 2017). Some researchers have concluded that mean differences in monitoring of girls and boys do not appear to affect the association between parental monitoring and later conduct problems (Keijsers et al., 2010; Kerr et al., 2010; Walters, 2019), nor the association with internalising problems like depression and low self-esteem (Hamza & Willoughby, 2011; Kerr & Stattin, 2000).

## Gendered parenting of LGBTQI + and gender non-conforming adolescents

To date, little research has specifically addressed gendered parenting of LGBTQI + and/or gender non-conforming adolescents. Much of the current literature either specifically excludes this adolescent population or does not report demographic information about participants beyond age and gender (typically reported as simply male or female). As such, it is difficult based on the current literature to examine how parenting practices impact this group of adolescents. That is, current sampling and research methodologies typically do not allow for comparative analysis of outcomes between groups of heterosexual, gender conforming adolescents and LGBTQI + and/or gender non-conforming adolescents. Despite these challenges we can draw on the body of work on parental acceptance, rather than on parenting practices specifically, to speculate on how gendered parenting may occur, and effects on LGBTQI + and gender non-conforming adolescents. For example, gendered parenting of adolescents may be exhibited in the degree of acceptance of gender non-conformity. Adolescence (particularly late adolescence) and early adulthood are associated with changes in sexual identity and gender presentation (Hasmanová-Marhánková, 2019; Stewart et al., 2019). For young women in particular, this period may be associated with multiple changes to their self-identified sexual orientation (Campbell et al., 2021). Gendered parenting may therefore be particularly problematic for adolescents who identify LGBTQI+ (regardless of whether they have ‘come out’ or not), due to the likelihood that these individuals do not conform with typical gendered expectations or beliefs parents may hold (Anderson, 2020). For example, in a sample of 6–12-year-old children, gender non-conformity was concurrently associated with greater behavioral and emotional problems, with a stronger association seen in individuals whose parents endorsed gender stereotypes (MacMullin et al., 2021). Furthermore, stigma is often reported by LGBTQI + and gender non-conforming individuals, and evidence suggests that parental stigma can be perceived as worse than other forms of stigma (Surace et al., 2020).

For both gender conforming and non-conforming LGBTQI + adolescents, gendered parenting and gender-typical expectations may be perceived as parental rejection (Mills-Koonce et al., 2018), which has been found to have a range of negative emotional and behavioral outcomes for adolescents (Ali et al., 2015). For gender non-conforming adolescents, gendered parenting may result in transgender or gender non-conforming adolescents not being ‘allowed’ to present in accordance with their internally felt gender (Warner et al., 2021). Support for an adolescent’s desired presentation (including their appearance, pronouns, name) may be an issue both for adolescents who do not identify with the gender they were assigned at birth, and for those who do not feel comfortable with a ‘standard’ gendered appearance (e.g., clothing style, use of makeup etc.; Abreu et al., 2019). Thus, attempts at parental control over gender identity and sexual orientation, and disapproval of gender non-conforming behavior or appearance may result in negative mental and physical health outcomes for adolescents (Mills-Koonce et al., 2018).

Gender policing is an area of research that has begun to receive attention in investigations of parental acceptance of LGBTQI + adolescents. Gender policing is associated with parental control and relates to the degree of freedom an adolescent has to express their gender identity based on expected social norms about gender (Bebes et al., 2015). In cross-sectional research, higher rates of parental gender policing have been found to be associated with poor mental health outcomes in adolescents generally (Bebes et al., 2015), and higher rates of substance use, depression, and anxiety among gender non-conforming adolescents and children (Bauermeister et al., 2017; Warner et al., 2021). Adolescents experience similarly negative mental health and social outcomes when they experience parental rejection after disclosing, or ‘coming out’ about their LGBTQI + identity, including higher rates of depression, attempted suicide, illicit drug use (Ryan et al., 2009), and increased risk of homelessness (James et al., 2016; Mills-Koonce et al., 2018). Parental rejection after coming out has also been found to be associated with poorer health outcomes for young adults (Newcomb et al., 2018). Research suggests that sexual health outcomes, including unsafe sex practices and rates of sexually transmitted infections, are poorer for adolescent gay or bisexual males who have low parental support (Garofalo et al., 2008), particularly low maternal support (Glick & Golden, 2014). Some evidence even suggests, perhaps counterintuitively, that high levels of parental control and monitoring are associated with higher rates of unsafe sex practices in young homosexual males (LaSala, 2015; Thoma & Huebner, 2014). This may occur due to reduced communication and education opportunities regarding healthy relationships and safe sex practices from parents, and a perceived need to conceal sexual orientation or behavior from parents.

In contrast, parenting that is supportive and accepting of gender non-conforming behavior is associated with positive outcomes for LGBTQI + adolescents (Warner et al., 2021). Research with younger children suggests that when parents allow their child to present and behave in gender non-conforming ways, LGBTQI + children aged 3–12 years have the same mental health outcomes as children in this age group who are not gender diverse or LGBTQI+ (Olson et al., 2016; Warner et al., 2021). Moreover, parental support of transgender adolescents is concurrently associated with greater life satisfaction, and a lower perception of their own transgender identity as burdensome (Simons et al., 2013).

## Conclusion and future directions

Overall, the limited research with adolescents indicates that there is likely to be continuity from childhood into adolescence in parents’ differential treatment and expectations of their children based on gender. However, more research is needed to better understand how parenting expectations and behaviors might differ for male and female adolescents, how it influences adolescent gender-typed behavior and attitudes, and the impact this has on psychosocial outcomes during this critical developmental stage. The following are key considerations for future research into gendered parenting of adolescents and its impact on adolescent outcomes.

### Longitudinal research is needed that considers the increasing reciprocity in parent-adolescent relationships

Much of the current research employed cross-sectional or short-term longitudinal designs. Research should focus on evaluating whether gendered parenting has longer-term impacts on adolescents, particularly as they transition into adulthood, across a broader range of health outcomes (e.g., physical activity, body image, sexual health), psychosocial outcomes, such as mental health (e.g., anxiety, depression, antisocial behavior) and relationships (e.g., preparation for sexual relationships, involvement in healthy relationships), and academic and employment outcomes. Importantly, this research should be driven by a conceptual framework (e.g., Endendijk et al., 2018) that considers the mechanisms by which gendered parenting influences psychosocial development. An important mediator, given the findings reported in this review, is likely to be adolescent gendered attitudes and behavior, such that the association between gendered parenting and psychosocial outcomes is at least partially mediated by adolescent gender development.

Longitudinal research that employs both parent and adolescent reports would also enhance our understanding of the reciprocal influence between parents and their adolescents. Research with LGBQTI + adolescents indicates that in well-functioning families, there is a shift from parental confusion and negativity at initial disclosure of LGBQTI + identity, to eventual acceptance and support of the young person (Warner et al., 2021). Thus, within a warm, connected and supportive parent-adolescent relationship, the adolescent has the potential to shift strongly held values about gender identity (and sexual orientation) in their parents. Because of the affectionate bond with their adolescent child, parents may be pushed to re-evaluate their beliefs about gender roles, gender identity or sexual orientation in a way that could not be achieved by even the most effective of public health campaigns. Thus, adolescents may have the capacity to affect positive change in their parents, which is an outcome that has not yet been empirically evaluated.

### Research is needed in diverse family, cultural and country contexts

Future research should be embedded within an ecological framework, by taking account of contextual factors that may moderate the ways in which gendered parenting is experienced by adolescents. Proximal factors likely to affect the relationship between gendered parenting and adolescent outcomes include family composition and gender of siblings; gender identity and sexual orientation of parents and the adolescent; and peers and schools. More distal factors include cultural and community norms and expectations, and the media (both online and traditional).

Given that much of the current research focuses on families headed by cohabitating couples, future research that considers diversity in family circumstances and sub-systems within families is needed. This should include, for example, single-parent households, households where parents do not cohabitate, and other family and living situations. A further limitation of most research on parents’ differential treatment of daughters versus sons is the reliance on between-family comparisons, that is, comparisons of boys in one group of families with girls in another group of families. Within-family comparisons of girls’ and boys’ experiences in the same families provide a stronger test of gender differences and a clearer understanding of the family circumstances within which gendered differential treatment emerges. Furthermore, we know very little about gendered parenting and its effects in cultural, political, and economic contexts in which gender disparities and inequalities are more apparent. Thus, future research on gendered parenting is needed in diverse cultural contexts. Research should also be performed within non-Western cultures, and cultures where multigenerational parenting is the norm, particularly given the potentially influential role of grandparents on adolescent development (Sadruddin et al., 2019; Yorgason et al., 2011).

### Research is needed that takes account of diversity in gender identity and sexual orientation

Diversity in gender identity and sexual orientation of parents and adolescents is largely absent from the current literature. Most research assumes that adolescents and their parents are heterosexual and cisgender. Thus, collection of sexual orientation and gender identity (SOGI) data should become routine. Moreover, future research should be designed to understand the interaction between gendered parenting practices (and associated outcomes) with gender identity and/or sexual orientation in adolescents, their parents, and other important caregivers. Future research is also needed to differentiate between acceptance of gender non-conforming behavior in LGBTQI + adolescents compared to acceptance of sexual orientation, given that many LGBTQI + adolescents present in a gender-conforming manner, while others do not. Finally, since much of the research on LGBTQI + and/or gender non-conforming adolescents has been directed at either gay or bisexual males, or transgender adolescents who were assigned male at birth (Newcomb et al., 2018), research is needed to better understand the health and psychosocial impact of gendered parenting and parental acceptance for female adolescents, or transgender adolescents who were assigned female at birth (McKay & Watson, 2020). It may be that improved parental support for gender identity and/or presentation during adolescence could improve outcomes for such individuals.

## Concluding thoughts

The adolescent period is increasingly recognised by researchers and policymakers as a critical intervention point for improving the overall health, mental health, and psychosocial outcomes of the community (Catalano et al., 2012) and specifically when it comes to issues of gender equality (Blum et al., 2017). This paper has provided an overview of the evidence base on gendered parenting of adolescents, with a particular focus on the effects of differential treatment of boys versus girls on adolescent psychosocial development. While the evidence is still building, research indicated that gendered parenting of adolescents exists and may have implications for adolescent gender role attitudes and preferred interests and activities even in egalitarian societies with higher rates of gender equality. However, much more research is needed to evaluate whether and how gendered parenting differentially effects male and female adolescents. Our review has highlighted a paucity of literature that moves away from the traditional two-parent family dynamic, noting the need for parenting research examining diverse families and a recognition of differing sociocultural contexts. In addition, in an environment of evolving attitudes towards gender identity, this review has identified the need for research that foregrounds diverse gender identities and sexualities. Within contemporary sociocultural frameworks, gender inequality is a driver of social change. Addressing the omissions in the research evidence would contribute to a shift towards greater gender equality, with potentially significant implications for the health and wellbeing of young people.

## Data Availability

Not applicable. Data sharing not applicable to this article as no datasets were generated or analysed during the current study.
